# Stab-Resistant Performance of the Well-Engineered Soft Body Armor Materials Using Shear Thickening Fluid

**DOI:** 10.3390/molecules27206799

**Published:** 2022-10-11

**Authors:** Rubin Wei, Bin Dong, Wen Zhai, Hui Li

**Affiliations:** 1 Key Laboratory for Liquid-Solid Structural Evolution and Processing of Materials, Ministry of Education, Shandong University, Jinan 250061, China; 2 Shandong Nonmetallic Materials Institute, Jinan 250031, China

**Keywords:** soft body armor, shear thickening fluid, stab resistance, fabric composites

## Abstract

Stab-resistant body armor can effectively prevent sharp instruments from attacking the protected parts and reduce the threat to human bodies. Shear thickening fluid (STF) is a kind of smart material with variable viscosity and its viscosity can change significantly with external stimuli. The soft and adaptive characteristics of STF provide a new idea for improving the performance of stab-proof materials. In this work, three kinds of soft anti-stabbing materials were designed and prepared with aramid, poly–p–phenylene benzodioxazole (PBO), and carbon fiber fabrics impregnated with STF. Quasi-static puncture tests and dynamic impact tests were conducted to compare the performance of different anti-stabbing structures. The results showed that the peak piercing force of the STF-treated fabrics in the puncture testing was greatly increased than that of neat samples. Against the D2 knife, the maximum impact load of STF/PBO fiber fabric was increased from 55.8 N to 72.9 N, increasing by 30.6%. Against the D3 spike, the maximum impact load of STF/aramid fabric was increased from 128.9 N to 254.7 N, increasing by 197.6%. The mechanical properties of fibers were important factors for the resistance to knives, and the fabric structure was the key point to bear the spike. Optical photographs of fabric fractures and scanning electron microscope analysis indicated that the STF effectively limited the slip of the fiber bundle when the tool penetrated the fabric, which played a positive role in maintaining the tightness and integrity of the fabric structure.

## 1. Introduction

In the modern world, with the imbalance of economic development, the gap between rich and poor around the world is widening, social contradictions are increasingly intensified, and the activities of terrorism, separatism and extreme religious forces are rampant in some regions, leading to political instability and frequent violent conflicts. The public security forces are the main force in fighting crimes and maintaining social stability and security. In order to effectively protect the life and safety of the police in the line of duty, the public security organs will uniformly equip them with various protective equipment, such as bullet proof armors, stab proof clothing, cut proof gloves, helmets, shields, and even riot clothing and explosive disposal clothing. As many countries strictly control guns and ammunition, the front-line police are threatened by piercing, hitting and chopping with sharp objects, such as knives and spikes. A large number of facts have proved that most of the injuries they suffered in violent incidents come from sharp objects, such as knives and sharp cones, rather than firearms. Therefore, the development of human protective equipment around the world focuses more on the design of stab-resistant armor.

Stab-resistant armor is a kind of equipment which can effectively prevent bladed weapons from attacking the protected parts and reduce the threat of stabbing the human body [1,2]. After years of research and development, clothing has gradually developed from hard armor to soft clothing. At the same time, the performance and environmental adaptability have been taken into account [3,4,5]. According to the properties of the materials, stab-proof clothing can be divided into metal stab-proof clothing and non-metal stab-proof clothing. The core materials of metal stab-resistant armor are alloy steel sheet, titanium alloy sheet, and high-strength aluminum alloy sheet. Metal stab-proof clothing has an excellent stab-resistant performance. However, due to its high hardness, large weight, and poor permeability, it is not conducive to the activities of the trunk muscles. The non-metal, stab-proof suits were made from non-metallic materials, such as engineering plastic sheets, high-strength and high-modulus polyethylene, aramid, and other fiber fabrics. Fiber composites could reduce the hardness of the materials, but still cannot meet the requirements of light-weight, low thickness, and softness in the military industry [6,7,8]. The stab-proof clothing is still a crucial problem affecting the office efficiency of police.

The shear thickening fluid (STF) is a kind of smart material with variable viscosity. Under the action of sudden shear, stretch, or impact force, the viscosity will change dramatically. In equilibrium state, STF shows macroscopically soft liquid state with low viscosity. Upon impact, its viscosity increases rapidly, and it behaves like a semisolid or even a solid [9,10,11,12,13]. In the process of liquid-solid transition, the STF dissipates the kinetic energy of impact to a larger area, so that it can resist impact effectively. The soft and adaptive characteristics of STF provide a new idea for improving the performance of stab-proof materials. Many attempts have been made to apply different kinds of STFs to composite with fibrous fabrics to produce flexible stab-proof armor. It has been proved that the STF can improve the stab-resistant performance of composites. Hassan et al. synthesized STF by dispersing silica nanoparticles (40%) into liquid polyethylene glycol (60%) with ultrasound irradiation. The Kevlar and Nylon fabrics were soaked in STF/ethanol solution to make STF/fabric composite. Quasi-static penetration tests showed that after STF treatment, the force of multiple layers of Kevlar fabrics was increased from 85 N to 573 N [14]. Hasanzadeh et al. conducted the puncture resistance tests of high modulus polypropylene (HMPP) fabric impregnated with STFs composed of fumed silica nanoparticles suspended in polyethylene glycol. The quasi-static puncture test showed that against a rounded tip penetrator, the maximum load of STF-HMPP fabric was increased from 1581.6 N to 2905.2 N, as compared to neat fabric [15]. Kang et al. treated Kevlar plain fabric with fumed silica-based STF and investigated the mechanical and stab-resistant properties. They found that 10 layers of STF-impregnated Kevlar composite fabric target against a spike supported significantly higher load ca. 350 N than neat target did ca. 100 N [16]. Yeh et al. quantitatively discussed the enhancement effect of STF on the stab-proof performance of STF/Kevlar composites, and the knife drop tower tests showed that the addition of the STF could improve the energy absorption by 20% [17]. Meanwhile, numerous studies have shown that the composition of STF [18,19,20], solid content [21,22], dispersed phase particle [21,22,23,24], as well as the molecular weight of dispersed medium [25] could influence the stab-resistant performance of composites. In our previous work, the influence of silica particle morphology on quasi-static and dynamic stab resistance of STF/fabrics was studied in detail, and the spherical particles have been proved to have greater application advantages to improve the material’s stabbing resistance [26].

Although there have already been many reports on the relationship between the properties of STF and the stab-proof property of composites, the effects of fabric type and weaving structure on the stab resistance of STF/fabric composites are rarely studied. In this paper, three kinds of soft anti-stabbing materials were prepared with different types of fiber fabric and shear thickening fluid, and the effects of the mechanical properties of fibers, as well as fabric structures, on the stab-proof performance of STF-impregnated fabrics under different knife and spike conditions were investigated.

## 2. Results and Discussion

### 2.1. Rheological Behavior of STF

**Figure 1:**
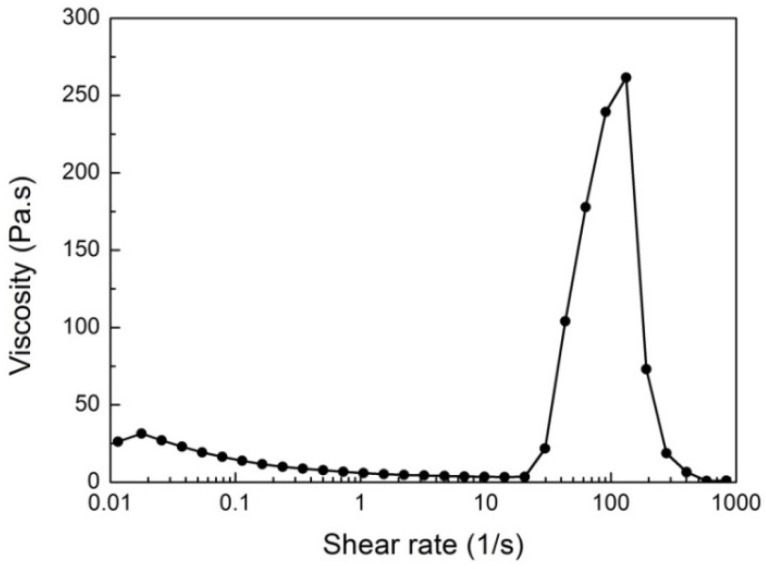
Viscosity versus shear rate of STF suspension.

The macroscopic performance of STF as a smart material is the variability of its viscosity with external stimuli, and the viscosity variation can be characterized by rheological parameters. The viscosity of the STF placed in the center of the parallel plate of the rheometer will change in real time when the shear rate changes. To measure the viscosity versus shear rate, the steady-state rheological test in the shear rate range of 0.1 s^−1^–1000 s^−1^ was conducted. The rheological property of STF was presented in Figure 1. As seen in Figure 1, the curve contained a shear-thinning area, a shear-thickening area, and a rapid decline area after reaching the peak viscosity within the testing limit. With the increase of the shear rate, the viscosity of STF gradually decreased and reached the minimum value of 3 Pa·s when the shear rate was 20.5 s^−1^. When the shear rate was higher than this critical value, shear thickening occurred, and the viscosity increased sharply until the maximum value of 270 Pa·s. After the viscosity exceeded this value, the STF became a macroscopic solid due to shear thickening. The STF distributed in the parallel plates of the rheometer was thrown out of the loading region by shear force, showing a rapid decrease in viscosity. According to the rheological parameters, the maximum viscosity of the STF was 90 times the minimum viscosity, showing an excellent thixotropic response effect.

### 2.2. Quasi-Static Behaviors of the STF/Fabrics

**Figure 2:**
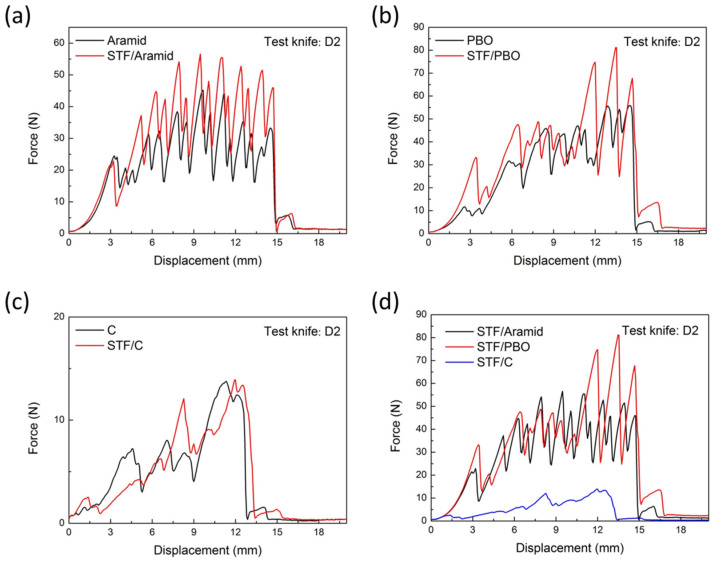
Force-displacement curves of neat and STF-treated fabrics penetrated with D2 tool. (**a**) Aramid-based fabrics; (**b**) PBO-based fabrics; (**c**) C-based fabrics; (**d**) STF-treated fabrics summary.

In order to characterize the stab resistance of STF/fabrics to a bladed instrument, the D2 tool was used to penetrate various fabric materials. The D2 tool was a standard knife with a continuous cutting edge (100 mm in length, 15 mm in width, and 2 mm in thickness), and the length of the cutting edge was 12 mm. The surface hardness of the tool body was 50–55 HRC. In the quasi-static impact test, the D2 tool penetrated into the fabrics at a speed of 200 mm/min. During this process, the force–displacement curves of the materials were recorded as shown in Figure 2.

The puncture force could be used to characterize the performance of the fabrics against puncture. As the cutting depth increased, the blade cut the fiber gradually, and the force between the fabric and the test tool was changed periodically with the destruction of the fabric structure. It was observed that, against the D2 knife, the STF-treated fabrics exhibited different protective behaviors compared with the neat targets. The peak piercing force, average value, and standard deviation of the fabrics against the D2 knife are shown in Table 1. The average peak piercing force of neat aramid fabrics was 44.2 N. The average peak piercing force of aramid treated with STF, however, increased to 50.1 N, which was 13.3% higher than that of the neat aramid fabric. The change of the puncture force of PBO fabrics before and after impregnating with STF also showed a similar pattern. After STF treatment, the maximum impact load of PBO fiber fabric was increased from 55.8 N to 72.9 N, with an increase of 30.6%. The increase of puncture force in carbon fiber fabric, however, was not obvious, and its puncture force was almost unchanged. In general, the puncture force of STF/PBO fabric composite was the largest after being impregnated with STF, indicating that the STF/PBO fiber composite had the strongest stab resistance.

**Figure 3:**
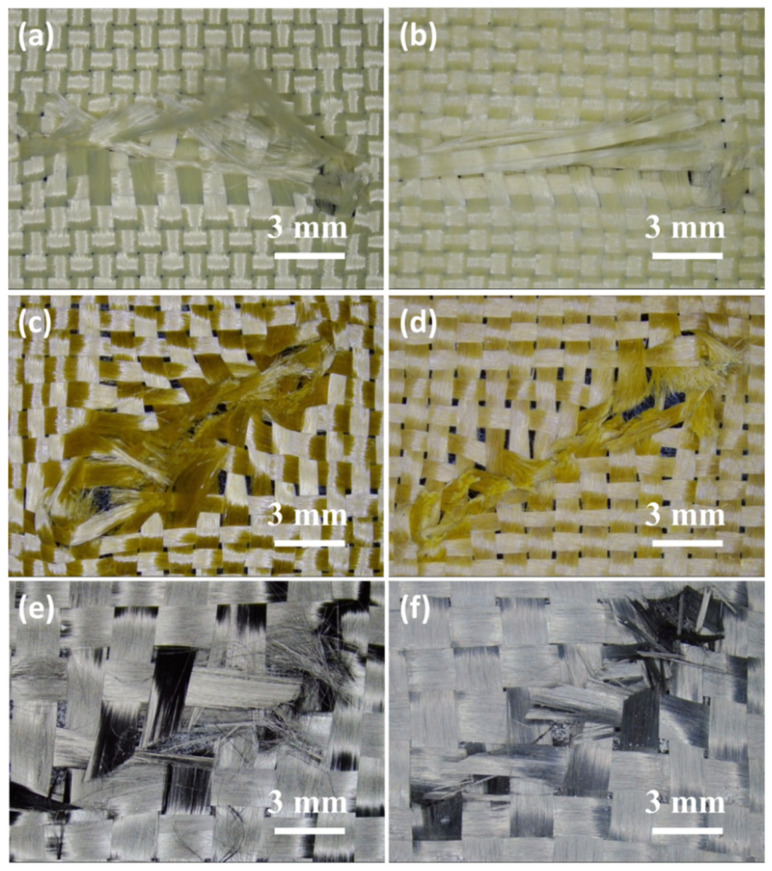
Optical micrographs of the damaged part of (**a**) aramid fabric; (**b**) STF/aramid fabric; (**c**) PBO fabric; (**d**) STF/PBO fabric; (**e**) C fabric; (**f**) STF/C fabric against the D2 tool.

Optical micrographs of the damaged part of the neat fabrics and STF/fabrics against D2 tool are shown in Figure 3. Obvious damage was found in the neat fabric, where the yarns were cut and fibrillated. The damage range of fabric structure at the fracture was large and the broken fibers were dispersed. The fractures of STF/fabrics were relatively neat, and the damage to fabric structure was not obvious. This might be attributed to the thixotropic hardening of the STF, resulting in the increased friction between fiber bundles, so that the broken fibers were bound in a smaller range, and the damage area was reduced. It can also be seen that under the same area, the numbers of fiber bundles of the three fabrics were not the same. Aramid fiber bundles were the densest, while carbon fiber bundles were the loosest. By combining the knife resistance of different fabrics (Figure 2d) with the tensile strength of the three fibers themselves, the STF/PBO fabric had the best knife-resistant performance. It can be speculated that the stab resistance of STF/fabrics against the D2 knife was closely related to the mechanical properties of the fibers themselves, while the fabric structure had no significant effect.

**Figure 4:**
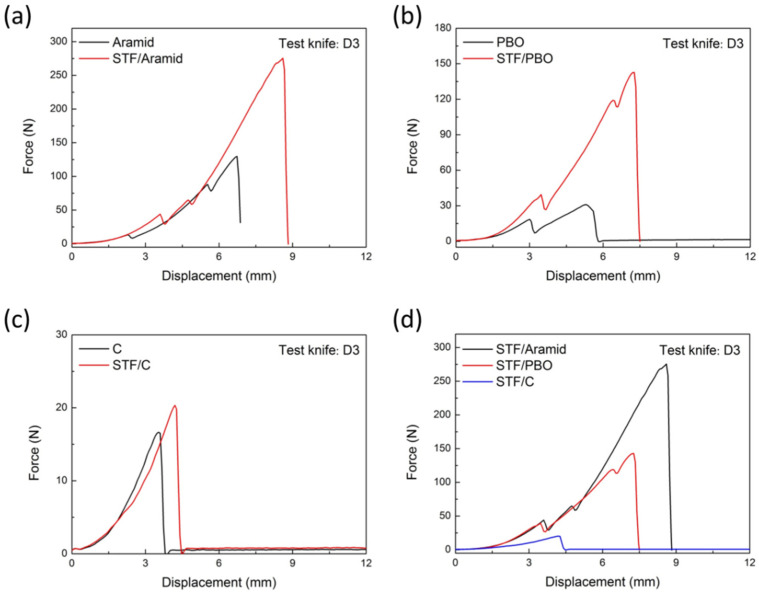
Force–displacement curves of neat and STF-treated fabrics penetrated with the D3 tool. (**a**) Aramid-based fabrics; (**b**) PBO-based fabrics; (**c**) C-based fabrics; (**d**) STF-treated fabrics summary.

In order to characterize the stab resistance of STF/fabrics to instruments with tips, the D3 tool was used to penetrate various fabric materials. The D3 tool was a small, sharp instrument with a tip and its shape was conical. The total length was 100 mm, the clamping end diameter was 4 mm, the puncture end diameter was 3 mm, the tip length was about 3 mm, and the surface hardness of tool body was 50–55 HRC. In the quasi-static impact test, the D3 tool penetrated into the fabrics at the same speed as the D2 tool. During this process, the force–displacement curves of the materials were plotted in Figure 4. It was observed that, against the spike impactor, all the STF-impregnated fabrics presented significantly larger puncture resistance force than the neat targets.

The peak piercing force, average value, and standard deviation of the fabrics against the D3 spike are shown in Table 2. After STF treatment, the maximum impact load of aramid fabric was increased from 128.9 N to 254.7 N, with an increase of 197.6%. The maximum impact load of PBO fabric was increased from 25.9 N to 135.4 N, increasing by 522.8%, and the maximum impact load of C fabric was increased from 12.2 N to 19.6 N, increasing by 60.7%. According to the maximum puncture force, the STF/aramid fabric had the best spike resistance. It was also suggested that the deformation for STF-impregnated fabrics was also greater than that of neat fabric. Taking aramid fabric as an example, the deformation for the neat aramid to the peak force was 6.7 mm. However, for the STF-treated sample, the value was increased up to 8.6 mm, presenting a better puncture resistance and deformation capacity. In addition, unlike the force–displacement curves of the D2 tool puncture, the curve of the D3 tool puncture did not fluctuate periodically. The D3 tool is a cylindrical spike with a pointed tip, which threatens the fabric only in the first few millimeters of puncturing. When the tool tip was completely passed through the fabric, only the smooth cylindrical part was left in contact with the fiber, and the friction force was almost negligible. Therefore, the puncture force reached its maximum value and then quickly dropped to zero.

**Figure 5:**
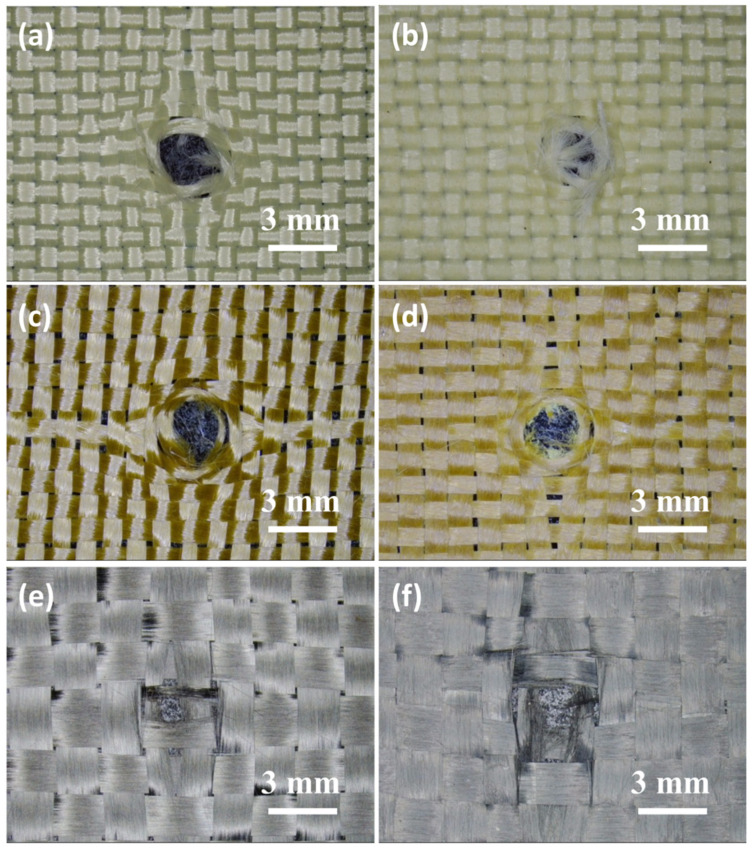
Optical micrographs of the damaged part of (**a**) aramid fabric; (**b**) STF/aramid fabric; (**c**) PBO fabric; (**d**) STF/PBO fabric; (**e**) C fabric; (**f**) STF/C fabric against the D3 tool.

Optical micrographs of the damaged part of the neat fabrics and STF/fabrics against the D3 tool are shown in Figure 5. For neat aramid and PBO fabrics, only a small number of fibers were broken during contact with the D3 tool, due to the small diameter of the tip of the tool, which moved forward along the fiber gap and pushed the fiber to one side. For STF-treated aramid and PBO fabrics, however, the fibers in the damaged area of the fabric were seriously broken and fibrillated, possibly due to the thixotropic hardening of the STF that restricted fiber movement, so that more fibers were directly involved in the stab resistance. The failure mode of carbon fiber fabric was slightly different with the above two kinds of organic fiber, its damage fracture area did not appear as a circular hole but there was some fiber fracture. This might be due to the brittleness of carbon fiber or the loose fabric structure, which decreased its stab-proof performance. The specific reasons should be further analyzed and verified. By combining the spike resistance of different fabrics (Figure 4d) with the tensile strength of the three fibers themselves, the STF/aramid fabric with the tightest weave structure had the best spike resistance. It could be found that the fabric structure was the critical factor influencing the stab-resistant performance of STF/fabric against the D3 spike, while the mechanical property of the fiber itself was not obvious.

### 2.3. Dynamic Impact Behaviors of the STF/Fabrics

When making body armor, one layer of stab-proof material is difficult to meet the needs of protection. Multiple layers of material are often assembled and applied to form a protective unit. To characterize the stabbing resistance of the fabric’s unit, dynamic drop tower stabbing impact tests of multilayer fabrics were performed at an energy of 6 J, 12 J, 18 J and 24 J, respectively. Aramid fabrics, with better quasi-static puncture resistance, were selected to characterize the dynamic puncture performance. To achieve a comparable weight, 11 layers of aramid fabric and eight layers of STF/aramid fabric were used to conduct the tests. To perform the tests, the tool dropped freely from a certain height, and the puncture energy could be controlled by adjusting the falling height. After the dynamic impact test, the puncture resistance of multilayer fabric was quantitatively characterized by the number of penetration layers.

**Figure 6:**
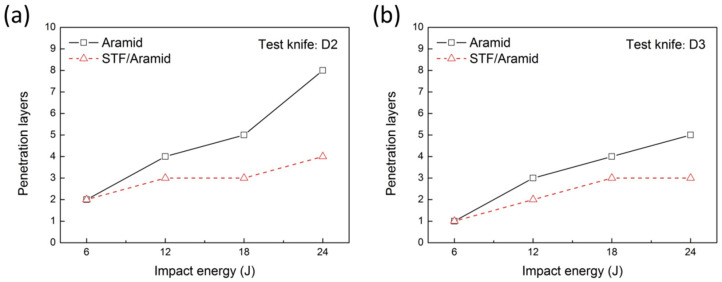
Relationship between fabrics’ penetration layers and impact energy. (**a**) D2 tool; (**b**) D3 tool.

The impact test results are summarized in Figure 6. It can be seen that the number of layers of all the penetrated fabrics was increased with the increase of puncture energy for both D2 and D3 tools. The fabrics treated with STF had less penetration layers than neat fabric, showing better stab resistance. With the increase of impact energy, this advantage was more significant. When the puncture energy was 24 J, the D2 tool penetrated eight layers of the neat aramid fabric, while only four layers of the STF/aramid fabric was penetrated. In addition, both the neat aramid fabrics and STF/aramid fabrics showed different dynamic impact resistance for different tools, and their protection against D3 tools was significantly better than D2 tools. According to the quasi-static puncture results of single-layer fabric, the maximum puncture force of neat aramid and STF/aramid on the D2 tool was 44.2 N and 50.1 N, and the maximum puncture force of neat fabric and STF/fabric on the D3 tool was 128.9 N and 254.7 N, respectively. The material showed better protection ability against the D3 tool under the quasi-static puncture condition. The dynamic impact test results of the multi-layer material further verified the conclusion.

### 2.4. Microstructures of the Neat and STF-Treated Fabrics

**Figure 7:**
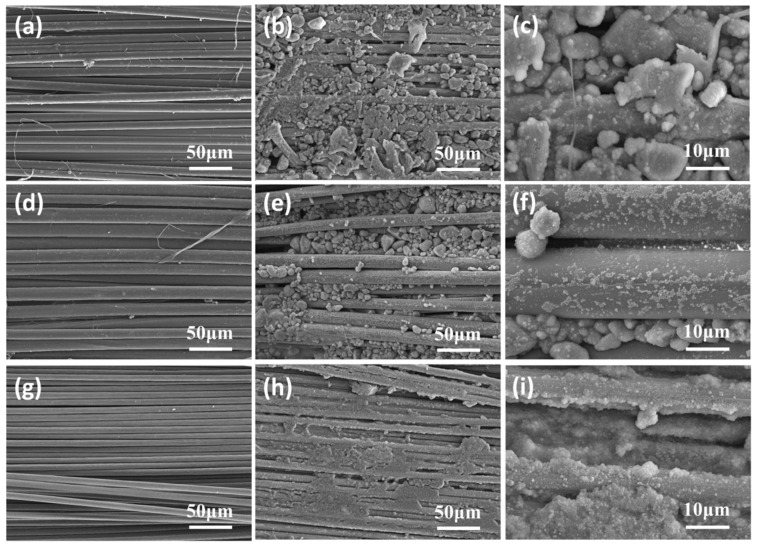
SEM images of neat and STF-treated fabrics. (**a**) Neat aramid fabric (1000×); (**b**) STF/aramid (1000×); (**c**) STF/aramid (5000×); (**d**) neat PBO fabric (1000×); (**e**) STF/PBO (1000×); (**f**) STF/PBO (5000×); (**g**) neat C fabric (1000×); (**h**) STF/C (1000×); (**i**) STF/C (5000×).

The microstructures of the neat and STF-impregnated fabrics were determined by scanning electron microscope (SEM), which was shown in Figure 7. The filaments in the neat yarns showed a smooth surface and the fibers were neatly packed together (Figure 7a,d,g). However, in filaments impregnated with STF, a large number of particles from the STF were found to be distributed in the gaps of the fiber bundles and on the fabric surface (Figure 7b,e,h). Further evidence from the higher magnification (Figure 7c,f,i) showed that these particles not only filled the surface of the fiber monofilaments but were also embedded in the gaps and fabric surfaces as blocks. Yarn pull-out and fabric windowing were two typical failure modes of the fabric in the process of puncture. When the knife penetrated the neat fabric, it first squeezed the bundle to one side and then passed through the fabric through the gap, where the protective properties of the fabric were not fully utilized. However, in the fabrics treated with STF, the composites had better continuity and integrity due to the relatively complete liquid phase structure formed by STF embedded in the gaps and fabric surfaces. The addition of STF effectively limited the slip of the fiber bundle when the tool penetrated the fabric, which played a positive role in maintaining the tightness of the fabric structure. This limitation was very helpful in improving the stab-proof performance of the fiber material. This might be the reason for the enhanced stab resistance of the above STF/fabrics.

## 3. Materials and Methods

### 3.1. Materials

The main materials used in this study were high-performance fiber fabrics and STF. The STF was a colloidal suspension composed of dispersed medium (polyethylene glycol with molecular weight of 200 g/mol, PEG200, Aladdin, Shanghai, China) and silica nanoparticles. The silica particles were mono-disperse spherical, with a particle size of about 200–300 nm, which were prepared using the Stöber method, through the hydrolyzation and condensation of tetraethylorthosilicate (TEOS, Aladdin, Shanghai, China) in an alkaline environment [27]. The fabrics used in this work were plain-woven fabrics comprised of p-aramid, poly-p-phenylene benzobisoxazole (PBO) and carbon yarns. The details of the fabrics are given in Table 3.

### 3.2. Preparation and Rheological Characterization of STF

The STFs were generated by intensive mixing of silica nanoparticles (70 wt. %) in PEG200 by mechanical agitation. In order to eliminate the bubbles introduced during the stirring process, the STF was vacuumed before use to ensure uniformity and stability. Rheological characterization of the STF was performed on a rheometer (TA, DISCOVERY HR-2, New Castle, DE, USA) with parallel plate principle, the shear rate was increased from 0.01 s^−1^ to 1000 s^−1^, and the corresponding viscosity was recorded. All measurements in this study were conducted at 25 °C.

### 3.3. Treatment of Fabrics with STF

To fabricate the STF/fabric composites, the STF was first diluted with anhydrous ethanol at 1:2 volume ratio of STF:ethanol to reduce the surface tension of the STF. The fabrics were cut into squares with sides of 10 cm and soaked in anhydrous ethanol to remove sizing agent before use. The impregnation of the fabrics was done by immersing the fabric specimens into the diluent for 10 min and then squeezing the excess liquid under a certain pressure. Finally, the drying process was carried out in an oven at 65 °C until the total weights of the fabric/STF assembly were unchanged. The details of the fabrics with STF are given in Table 4.

### 3.4. Quasi-Static Impact Tests

**Figure 8:**
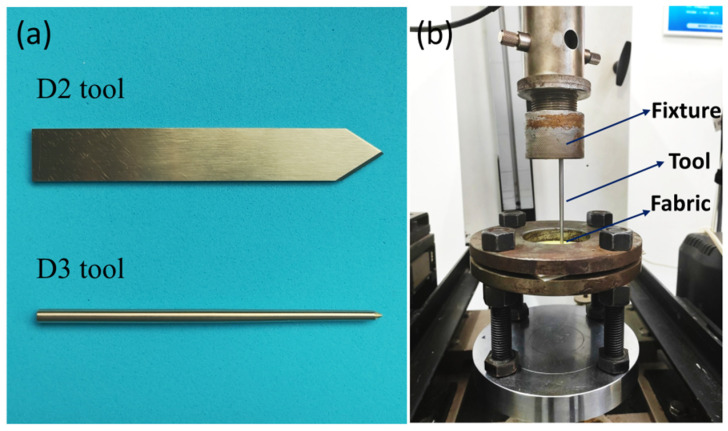
Test tools and equipment used in the quasi-static impact experiments.(**a**) Test tools; (**b**) Test fixture.

The quasi-static impact tests were performed on a universal tester (Instron 5966). Two typical test tools (D2, D3) were used in the quasi-static impact experiments. The D2 tool was a standard knife and the D3 tool was a spike, of which the size and shapes were made according to the Chinese Ministry of Public Security Standard, GA68-2019 “Stabbing Resistance of Personal Body Armor”. The tools and test fixture are shown in Figure 8. The fabric was tightly fixed by a ring-shaped clamp with an outer diameter of 150 mm and an inner diameter of 50 mm, and a layer of silicone rubber was covered on the upper and lower surfaces of the fabric to increase sliding resistance. Before the test, the tool was fixed on the universal testing machine. Then, the test tool impacted into the center of the fabric at a rate of 200 mm/min and the load-displacement diagram during the impact process was obtained. To improve the data consistency, three samples were tested for each material.

### 3.5. Dynamic Impact Tests

The dynamic impact tests were conducted on a drop tower puncture tester also according to the Chinese Standard, GA68-2019. The stab targets were placed on a multi-layer foam backing, as specified by the GA68 standard. The backing was laminated from the following materials: four layers of 6-mm-thick neoprene sponge, followed by one layer of 30-mm-thick polyethylene foam, and the bottom were two 6.5-mm-thick layers of rubber. In the dynamic impact test, the test tools were loaded to the drop mass and lifted to a fixed height. Then, the tool dropped freely into the sample, and the number of penetration layers was used to characterize the stab-resistant performance. In this work, the dropping energy was 6 J, 12 J, 18 J and 24 J, respectively.

## 4. Conclusions

In summary, the STF-treated fabrics presented much larger puncture resistance force than the neat targets in quasi-static puncture tests. Against the D2 tool, the maximum impact load of STF/PBO fiber fabric increased from 55.8 N to 72.9 N, increasing by 30.6%, which demonstrated an optimum knife resistance. Against the D3 tool, the maximum impact load of STF/aramid fabric increased from 128.9 N to 254.7 N, increasing by 197.6%, which was the best spike resistance material. The mechanical property of fibers was an important factor for the resistance to knife, and the fabric structure was the key point of the resistance to spike. The dynamic impact tests of the multiple layers of material showed that the number of penetrating layers decreased obviously after the addition of STF, which further confirmed the improvement of STF’s anti-stabbing performance.

## Figures and Tables

**Table 1:** Peak piercing force of different fabrics when punctured by D2 knife.

Samples	Tool	Peak Piercing Force (N)	Average (N)	Standard Deviation
P-aramid fabric	D2	45.1; 47.0; 40.5	44.2	3.3
STF/P-aramid fabric	D2	56.6; 46.2; 47.4	50.1	5.7
PBO fabric	D2	55.9; 63.2; 48.4	55.8	7.4
STF/PBO fabric	D2	81.1; 65.8; 71.7	72.9	7.7
Carbon fabric	D2	13.8; 14.3; 9.9	12.7	2.4
STF/Carbon fabric	D2	13.9; 11.5; 12.5	12.6	1.2

**Table 2:** Peak piercing force of different fabrics when punctured by D3 knife.

Samples	Tool	Peak Piercing Force (N)	Average (N)	Standard Deviation
P-aramid fabric	D3	129.9; 142.1; 114.6	128.9	13.8
STF/P-aramid fabric	D3	275.6; 254.4; 234.1	254.7	20.8
PBO fabric	D3	30.9; 20.1; 26.8	25.9	5.5
STF/PBO fabric	D3	142.7; 110.1; 153.4	135.4	22.6
Carbon fabric	D3	16.6; 9.6; 10.5	12.2	3.8
STF/Carbon fabric	D3	20.3; 18.5; 20.0	19.6	1.0

**Table 3:** Specification of the fabrics.

Samples	Areal Density (g/m^2^)	Fibre Specification	Fiber Tensile Strength (GPa)
P-aramid fabric	200	840 D	3.4
PBO fabric	206	1000 D	5.8
Carbon fabric	200	3 K	2.3

**Table 4:** Specification of the fabrics with STF.

Samples	STF Content (wt.%)	Areal Density (g/m^2^)
STF/P-aramid fabric	24.8	266
STF/PBO fabric	24.5	273
STF/Carbon fabric	27.0	274

## Data Availability

The data used to support the findings of this study are included in the article.
